# Supplementary material for: A Humanized Yeast Phenomic Model of Deoxycytidine Kinase to Predict Genetic Buffering of Nucleoside Analog Cytotoxicity
Source: Genes (Basel). 2019 Sep 30;10(10):770. doi: 10.3390/genes10100770 (PMC6826991; doi:10.3390/genes10100770)
Supplement: Supplementary file 1 [file genes-10-00770-s001.zip › Additional_Files/Additional_File7_GOTermHeatmaps.zip!/Additional_File7_GOTermHeatmaps/peptide_metabolic_process.pdf]

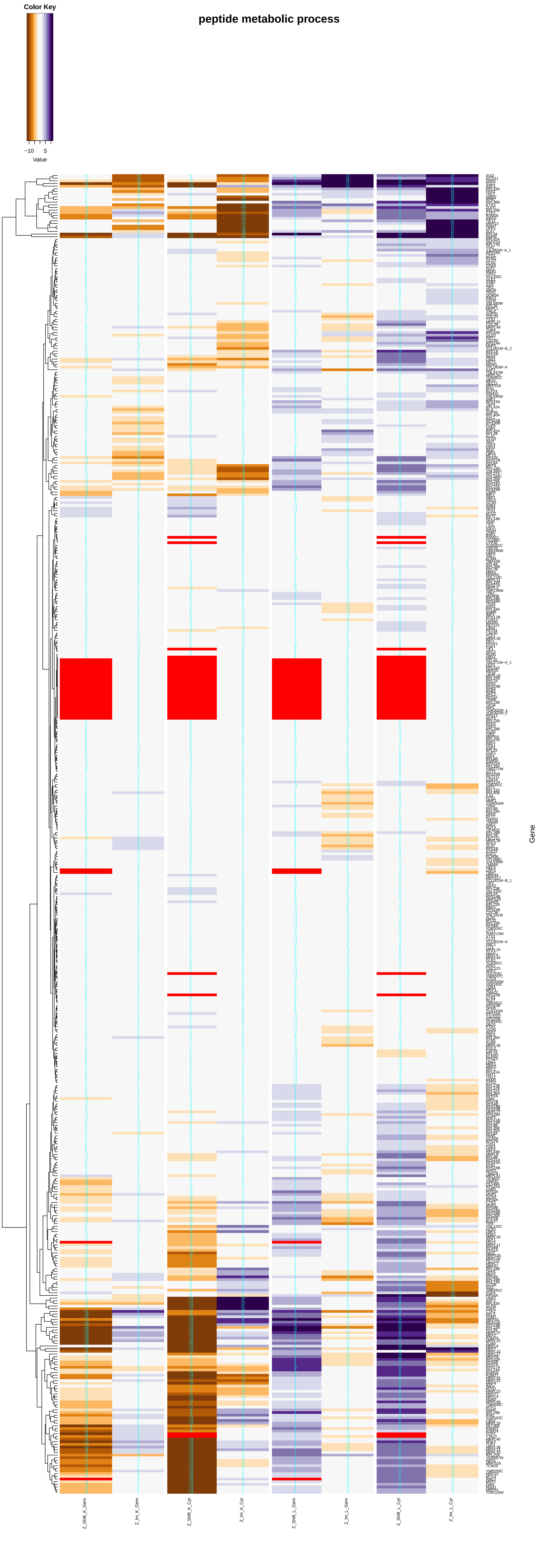
peptide metabolic process
Color Key
−10
5
Value
Z_Shift_K_Gem
Z_Im_K_Gem
Z_Shift_K_Cyt
Z_Im_K_Cyt
Z_Shift_L_Gem
Z_Im_L_Gem
Z_Shift_L_Cyt
Z_Im_L_Cyt
Gene

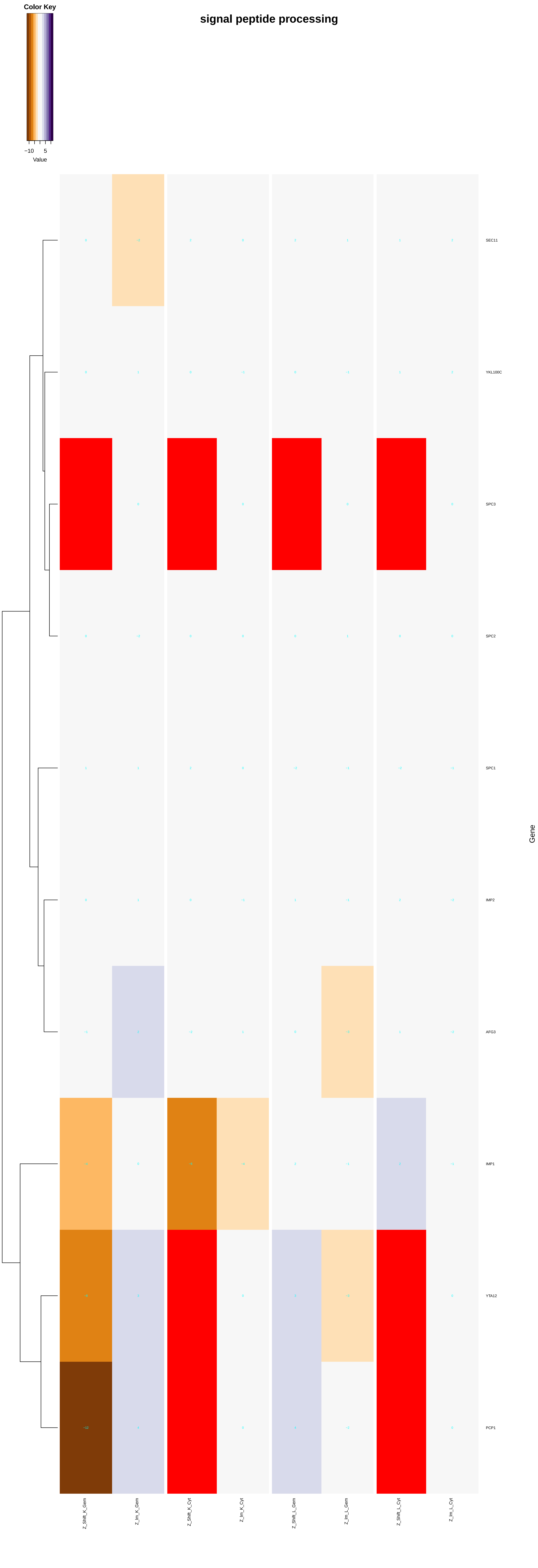
signal peptide processing
Color Key
−10
5
Value
SEC11
YKL100C
SPC3
SPC2
SPC1
IMP2
AFG3
IMP1
YTA12
PCP1
Gene
Z_Shift_K_Gem
Z_Int_K_Gem
Z_Shift_K_Cyt
Z_Int_K_Cyt
Z_Shift_L_Gem
Z_Int_L_Gem
Z_Shift_L_Cyt
Z_Int_L_Cyt

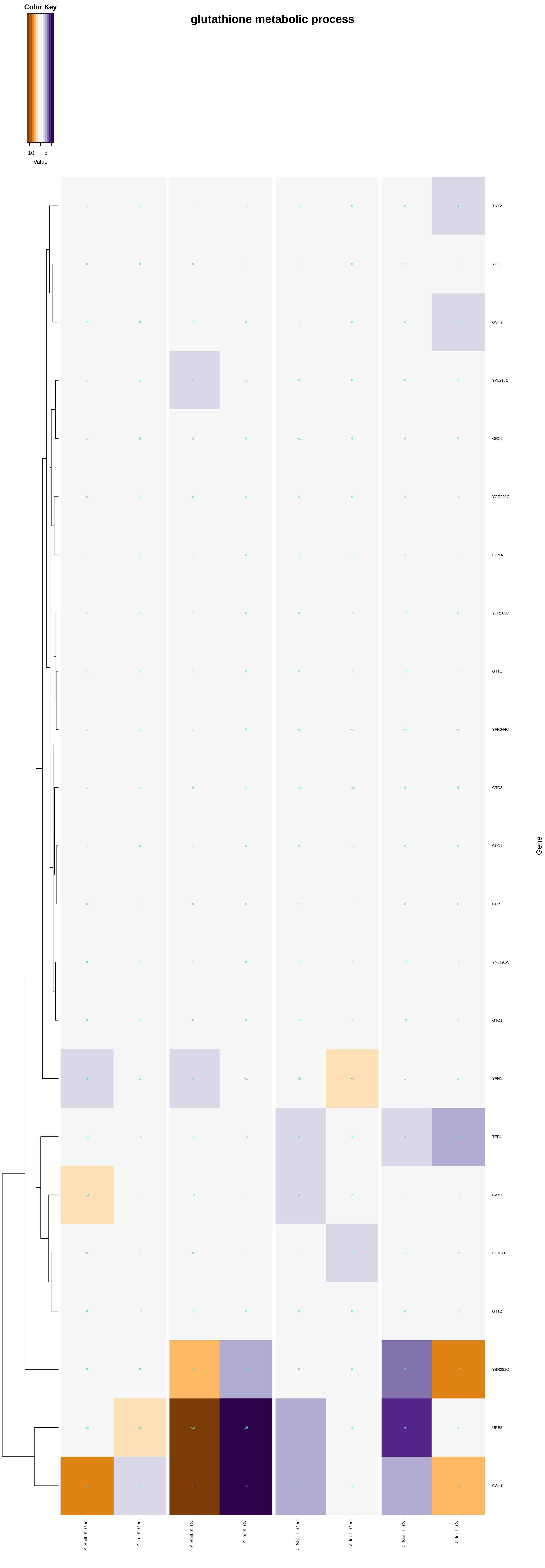
glutathione metabolic process
Color Key
−10
5
Value
TRX2
YCF1
GSH2
YKL215C
GRX2
YGR201C
ECM4
YER163C
GTT1
YFR044C
GTO3
GLO1
GLR1
YNL191W
GTO1
YFH1
TEF4
CAM1
ECM38
GTT2
YBR281C
URE2
GSH1
Gene
Z_Shift_K_Gem
Z_Im_K_Gem
Z_Shift_K_Cyt
Z_Im_K_Cyt
Z_Shift_L_Gem
Z_Im_L_Gem
Z_Shift_L_Cyt
Z_Im_L_Cyt

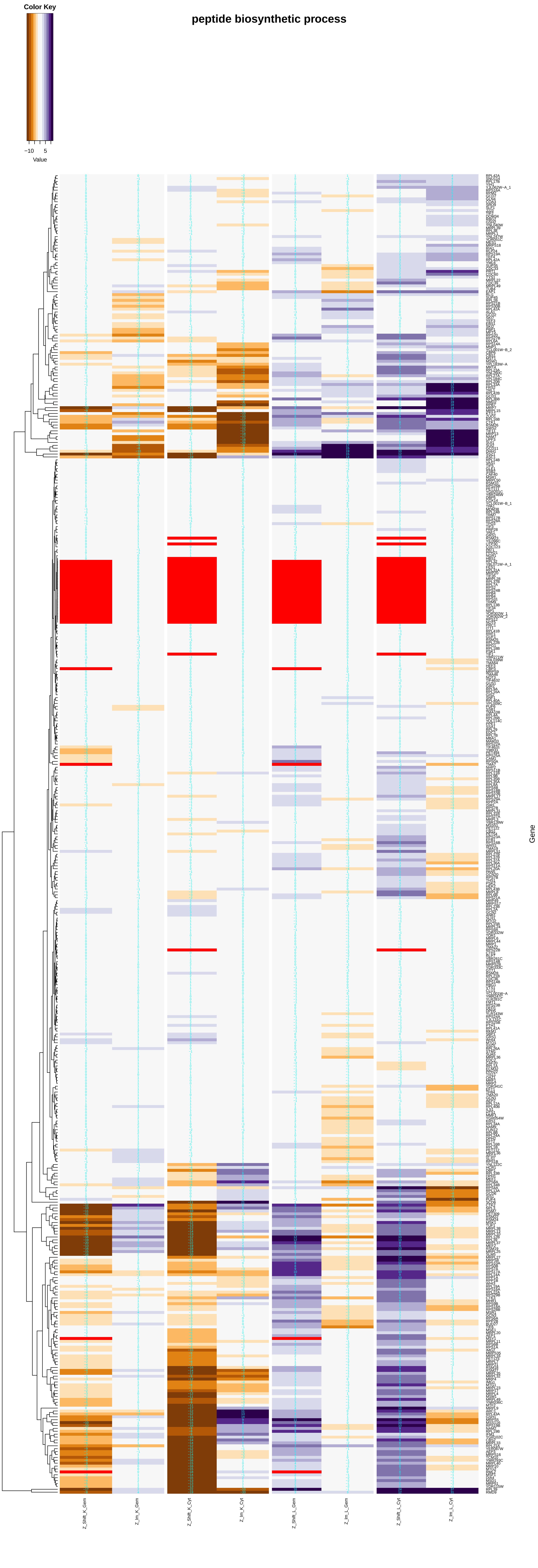

peptide biosynthetic process
Color Key
−10
5
Value
Gene
Z_Shift_K_Gem
Z_Im_K_Gem
Z_Shift_K_Cyt
Z_Im_K_Cyt
Z_Shift_L_Gem
Z_Im_L_Gem
Z_Shift_L_Cyt
Z_Im_L_Cyt

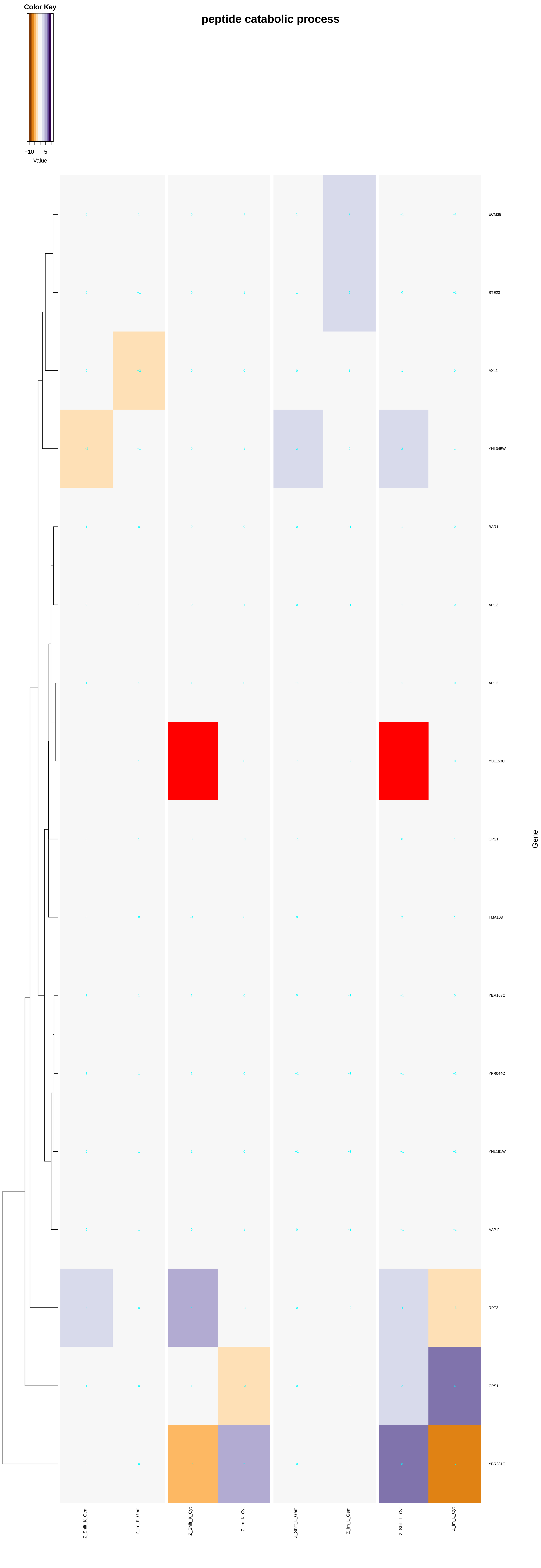
peptide catabolic process
Color Key
−10
5
Value
ECM38
STE23
AXL1
YNL045W
BAR1
APE2
APE2
YOL153C
CPS1
TMA108
YER163C
YFR044C
YNL191W
AAP1'
RPT2
CPS1
YBR281C
Gene
Z_Shift_K_Gem
Z_Im_K_Gem
Z_Shift_K_Cyt
Z_Im_K_Cyt
Z_Shift_L_Gem
Z_Im_L_Gem
Z_Shift_L_Cyt
Z_Im_L_Cyt

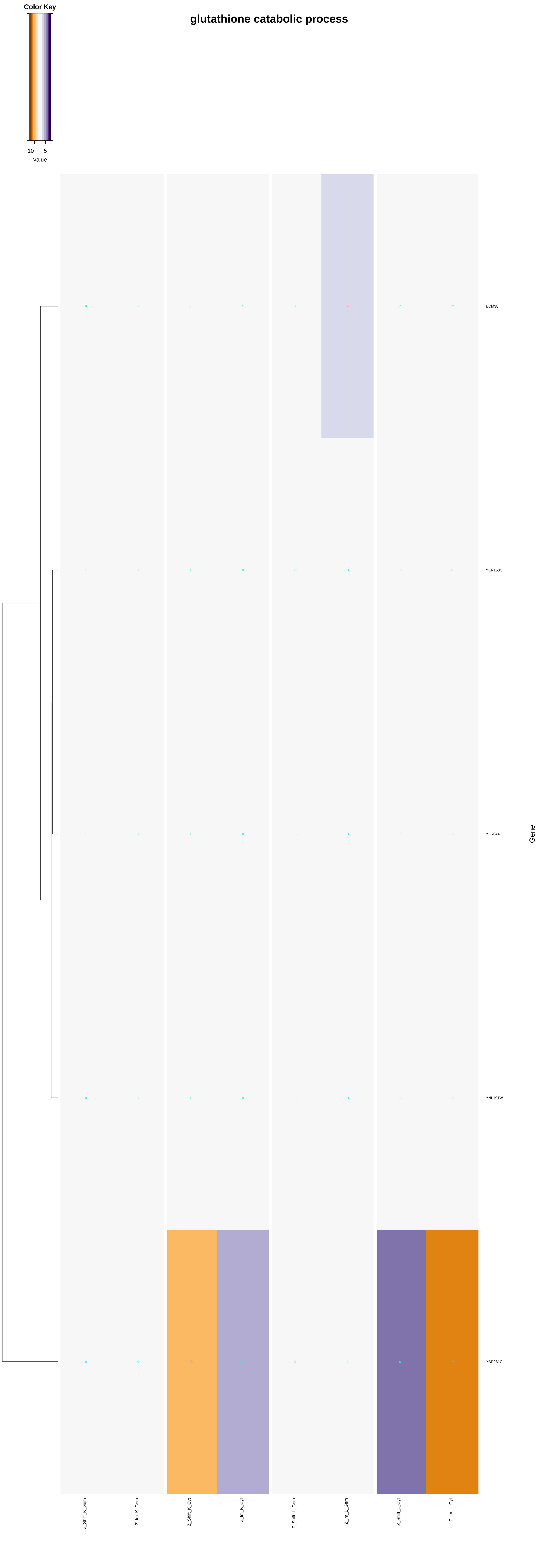
glutathione catabolic process
Color Key
−10
5
Value
ECM38
YER163C
YFR044C
YNL191W
YBR281C
Gene
Z_Shift_K_Gem
Z_lm_K_Gem
Z_Shift_K_Cyt
Z_lm_K_Cyt
Z_Shift_L_Gem
Z_lm_L_Gem
Z_Shift_L_Cyt
Z_lm_L_Cyt

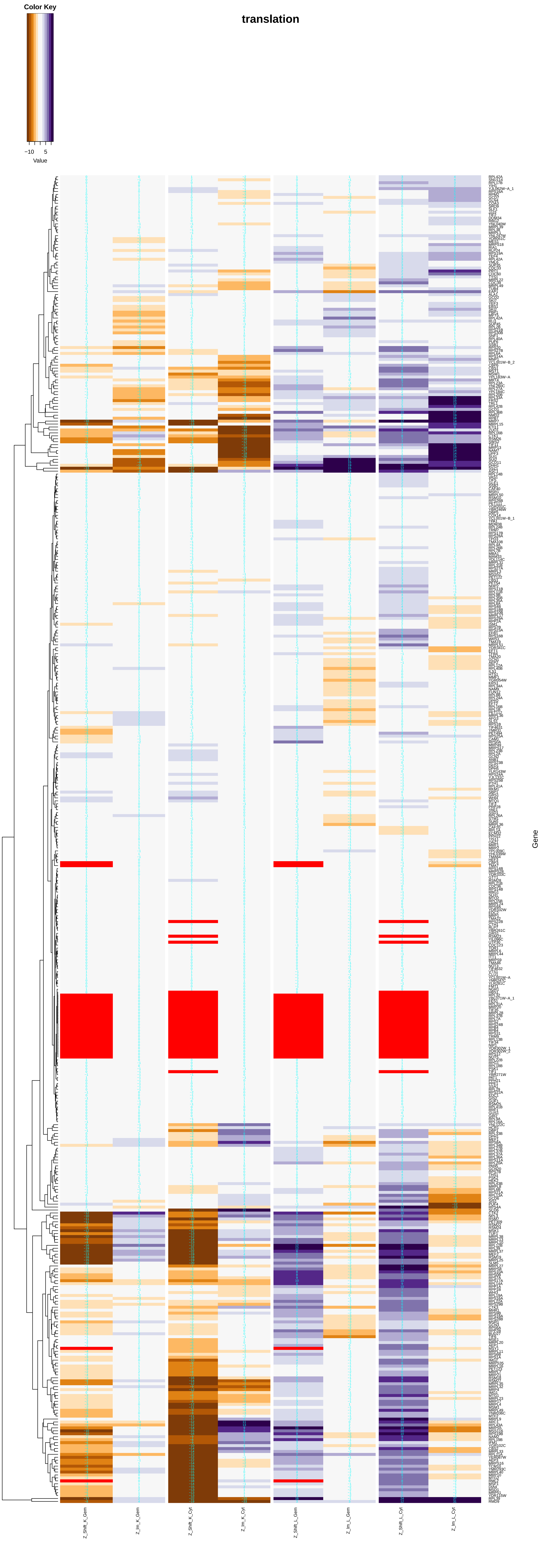
translation
Color Key
−10
5
Value
Z_Shift_K_Gem
Z_Im_K_Gem
Z_Shift_K_Cyt
Z_Im_K_Cyt
Z_Shift_L_Gem
Z_Im_L_Gem
Z_Shift_L_Cyt
Z_Im_L_Cyt
Gene

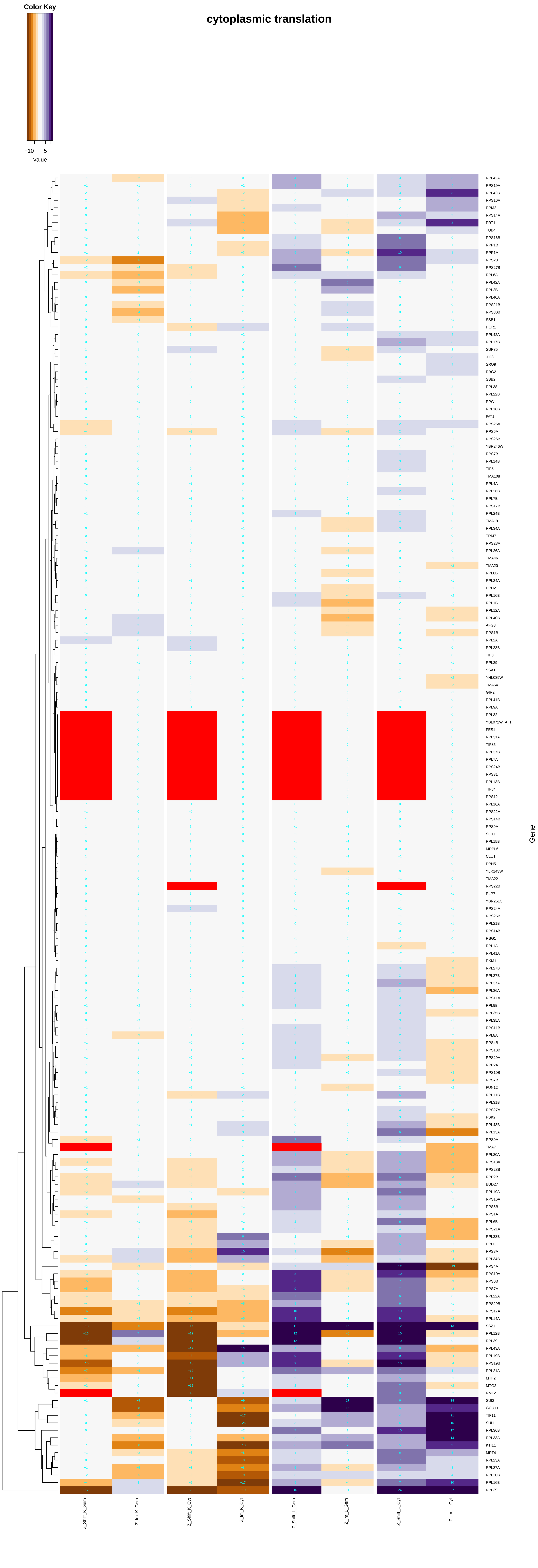

cytoplasmic translation
Color Key
−10
5
Value
Gene
Z_Shift_K_Gem
Z_Im_K_Gem
Z_Shift_K_Cyt
Z_Im_K_Cyt
Z_Shift_L_Gem
Z_Im_L_Gem
Z_Shift_L_Cyt
Z_Im_L_Cyt

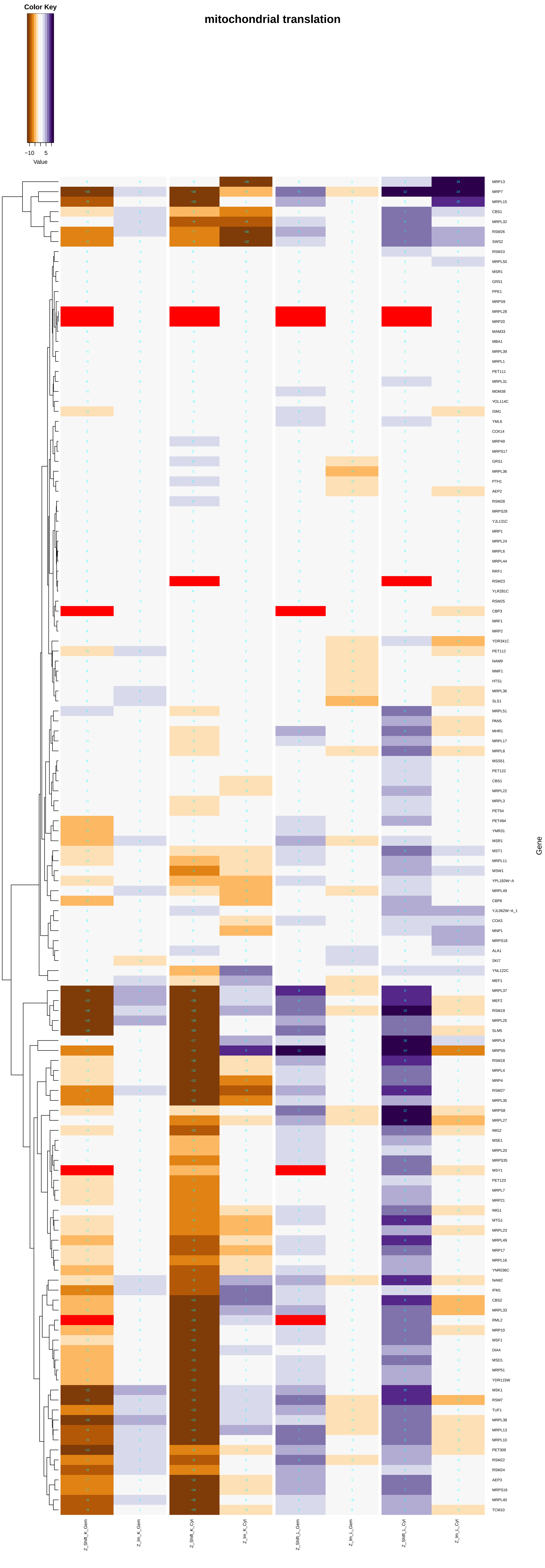

Color Key
−10
5
Value
mitochondrial translation
MRP13
MRP7
MRPL15
CBS1
MRPL32
RSM26
SWS2
RSM10
MRPL50
MSR1
GRS1
PPE1
MRPS9
MRPL28
MRP20
MAM33
MBA1
MRPL39
MRPL1
PET111
MRPL31
MDM38
YOL114C
ISM1
YML6
COX14
MRP49
MRPS17
GRS1
MRPL36
PTH1
AEP2
RSM28
MRPS28
YJL131C
MRP1
MRPL24
MRPL6
MRPL44
RRF1
RSM23
YLR281C
RSM25
CBP3
MRF1
MRP2
YDR341C
PET112
NAM9
MMF1
HTS1
MRPL36
SLS1
MRPL51
PAN5
MHR1
MRPL17
MRPL8
MSS51
PET122
CBS1
MRPL22
MRPL3
PET54
PET494
YMR31
MSR1
MST1
MRPL11
MSW1
YPL183W-A
MRPL49
CBP6
YJL062W-A_1
COA3
MNP1
MRPS18
ALA1
SKI7
YNL122C
MEF1
MRPL37
MEF2
RSM19
MRPL25
SLM5
MRPL9
MRPS5
RSM18
MRPL4
MRP4
RSM27
MRPL35
MRPS8
MRPL27
IMG2
MSE1
MRPL20
MRPS35
MSY1
PET123
MRPL7
MRP21
IMG1
MTG1
MRPL23
MRPL49
MRP17
MRPL16
YNR036C
NAM2
IFM1
CBS2
MRPL33
RML2
MRP10
MSF1
DIA4
MSD1
MRP51
YDR115W
MSK1
RSM7
TUF1
MRPL38
MRPL13
MRPL10
PET309
RSM22
RSM24
AEP3
MRPS16
MRPL40
TCM10
Gene
Z_Shift_K_Gem
Z_Im_K_Gem
Z_Shift_K_Cyt
Z_Im_K_Cyt
Z_Shift_L_Gem
Z_Im_L_Gem
Z_Shift_L_Cyt
Z_Im_L_Cyt
